# Emergency total gastrectomy for gastric perforation secondary to metastatic lobular breast cancer: a case for aggressive surgical intervention in select patients

**DOI:** 10.1093/jscr/rjaf387

**Published:** 2025-06-06

**Authors:** Amirpasha Rafizadeh, Tze Wei Wilson Yang, Simon Nazaretian, Richard Chen

**Affiliations:** Department of Medicine, Nursing and Health Sciences, Monash University, Wellington Rd, Clayton, VIC 3800, Australia; Department of Oesophago-Gastric and Bariatric Surgery, Alfred Hospital, 55 Commercial Rd, Melbourne, VIC 3004, Australia; Department of Anatomical Pathology, Alfred Hospital, 55 Commercial Rd, Melbourne, VIC 3004, Australia; Department of Oesophago-Gastric and Bariatric Surgery, Alfred Hospital, 55 Commercial Rd, Melbourne, VIC 3004, Australia

**Keywords:** gastrectomy, perforation, metastatic breast cancer

## Abstract

Breast cancer remains a predominant contributor to cancer-related morbidity and mortality among women worldwide. Invasive lobular carcinoma (ILC), the second most prevalent histological subtype, exhibits a distinctive propensity for diffuse infiltration and distant metastases, including rare involvement of the gastrointestinal tract. We describe a case of acute gastric perforation secondary to metastatic ILC in a 67-year-old woman with recurrent Luminal A disease. Her prior treatment included wide local excision, adjuvant chemoradiotherapy, and salvage mastectomy. Emergent imaging revealed gastric perforation with diffuse intraperitoneal fluid. Following multidisciplinary discussion and in accordance with the patient’s wishes, an R1 total gastrectomy was performed with palliative intent. The patient recovered well, despite a minor duodenal stump leak managed conservatively. She was subsequently discharged for ongoing oncological management. To our knowledge, this is the first reported case of emergency total gastrectomy for ILC-related gastric perforation, highlighting the potential role of aggressive surgical intervention in select individualized metastatic presentations.

## Introduction

Breast cancer is the most common malignancy among women and remains a leading cause of cancer-related morbidity and mortality worldwide [1]. Invasive lobular carcinoma (ILC), the second most common histological subtype, accounts for 5%–15% of invasive breast cancers. Compared to invasive ductal carcinoma (IDC), ILC is characterized by a diffuse infiltrative growth pattern, predisposing it to distant metastases [2]. While ILC commonly spreads to bone, lung, and liver, gastrointestinal (GI) involvement is rare, with gastric metastasis reported in only 0.2%–0.7% of cases [3].

We present a case of known gastric metastatic ILC presenting with acute perforation, managed successfully with emergency total gastrectomy.

## Case presentation

A 67-year-old woman presented in August 2024 with the acute onset of severe abdominal pain associated with dyspnea. Her history included T2N1 left-sided breast ILC treated with lumpectomy and adjuvant chemoradiotherapy in 1999. She had a recurrence in 2020 (ER positive, PR negative, HER2 negative, low Ki-67) involving the contralateral axillary node, requiring bilateral mastectomy and axillary clearance with right axillary radiotherapy. Despite a significant family history of early-onset breast cancer (sister, mother, and maternal grandmother), genetic testing via the Breast, Ovarian, Prostate, and Pancreatic cancer (BOPP) panel revealed no pathogenic variants. Most recently, she was diagnosed with ILC metastasis to the stomach and treated with hormonal therapy and a CDK4/6 inhibitor.

**Figure 1:**
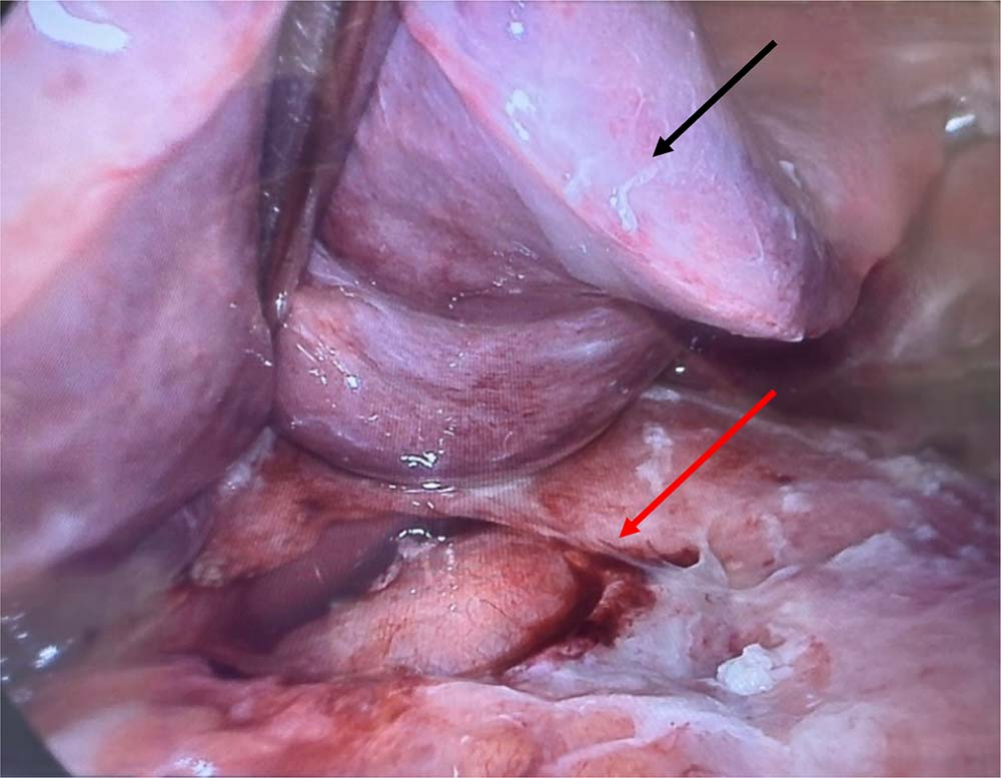
Intraoperative image with liver (black/upper arrow) retracted superiorly, with large tumor (red/lower arrow) surrounding the lesser curvature, anterior antrum of the stomach, and porta hepatis, accompanied by fibrin deposits.

An initial computed tomography (CT) scan revealed diffuse thickening of the gastric wall, free intraperitoneal gas and fluid, and a suspected perforation of the anterior/superior stomach wall. She underwent a diagnostic laparoscopy and washout. Intraoperatively, a large infiltrative tumor was found involving the lesser curvature and anterior antrum, with fibrin deposits (Fig. 1). An on-table gastroscopy only demonstrated a gastric ulcer at the anterior wall of gastric antrum close to incisura, with no definitive perforation site identified. It was postulated that the perforation site may have sealed spontaneously. Thorough abdominal washout was performed, and surgical drains were placed.

**Figure 2:**
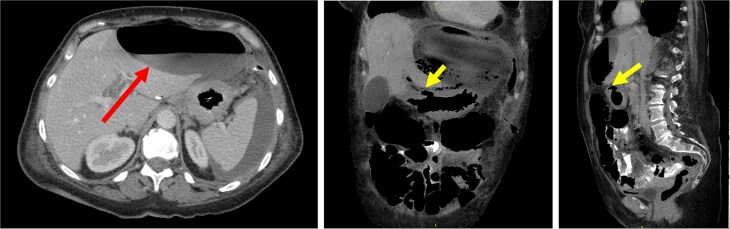
CT images demonstrated a large, thin-walled anterior epigastric collection with an air-fluid level (long/red arrow) measuring 10.8 × 5.8 × 16.4 cm, positioned anterior to the left hepatic lobe and the gastric body. This collection appeared to communicate with the suspected perforation site (short/yellow arrows). Additionally, loculated fluid pockets were observed around the spleen and the left upper quadrant bowel loops, along with interspersed free fluid and gas locules likely leaking from the stomach.

**Figure 3:**
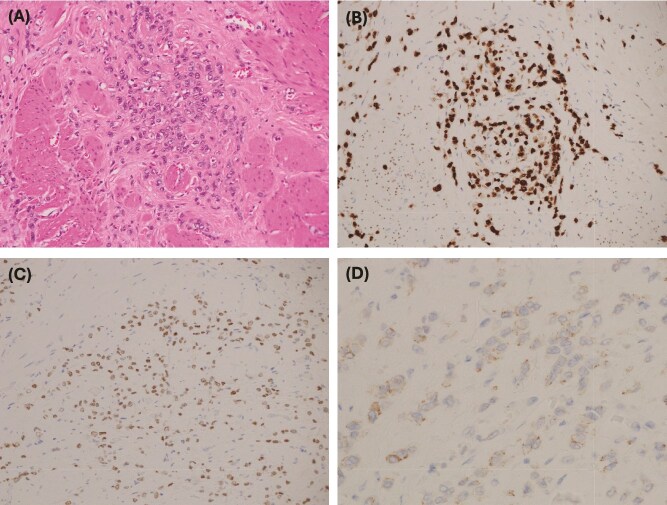
(A) Medium power: atypical cells with a small amount of cytoplasm and hyperchromatic nuclei with vesicular chromatin and small nucleoli. (B) GATA 3 immunohistochemistry: strong positive nuclear staining, which in the context of CK7 positivity and CK20 negativity is in keeping with metastatic carcinoma of breast origin—supported by positive estrogen receptor staining. (C) Estrogen receptor IHC: 2+ staining. PR and HER2 were negative. (D) E-cadherin IHC: weak patchy staining is negative and is in keeping with lobular differentiation—explains the poorly cohesive nature of the tumor cells, which infiltrate as single cells and single files, and cords rather than well-developed nests and ductal formations.

Postoperatively, the patient experienced persistent abdominal pain and rising inflammatory markers. Repeat CT showed an enlarging intra-abdominal collection suggestive of ongoing leakage (Fig. 2). She expressed a strong desire for life-saving treatment, despite her diagnosis of advanced metastatic disease. After extensive multidisciplinary discussion, an emergency open total gastrectomy with Roux-en-Y reconstruction was performed. This was undertaken with a palliative R1 resection intent. The operation was technically challenging because the stomach had extensive tumor invasion to the retroperitoneum, porta hepatis, as well as splenic hilum. Despite a difficult resection, the case progressed well. The resected specimen showed metastatic poorly differentiated diffuse gastric adenocarcinoma of breast origin with features of pleomorphic ILC, involving the proximal resection margin and four out of seven lymph nodes with metastatic carcinoma (Fig. 3).

Post-operative recovery was largely uneventful with successful gradual diet upgrade and resolved sepsis. There was a low volume bile leak from the duodenal stump noted day 7 postoperatively, which was managed conservatively with the peri-duodenal drain placed at the time of surgery. The bile leak resolved spontaneously. The drain was removed prior to her discharge from her 4-week patient stay. Patient remained well and was tolerating a normal diet at her follow-up 3 months post-discharge. She was planned to start second-line chemo/immunotherapy with her medical oncologist.

## Discussion

ILC breast cancer has a well-established tendency to spread to distant organs. The diffuse growth pattern of ILC may contribute to its metastatic behavior, yet gastric involvement is an uncommon manifestation. The reported incidence of breast cancer metastasis to the stomach ranges from 0.1% to 6%, though autopsy series suggest a higher rate, estimated at 5% to 18% [4]. This rarity is further compounded by the fact that gastric metastases often mimic primary gastric cancers or benign GI conditions, such as gastritis, posing a significant diagnostic challenge.

Gastric metastasis typically presents as diffuse linitis plastica-like infiltration [4]. In these cases, the presenting symptoms often included nonspecific GI complaints, such as nausea, vomiting, abdominal pain, and GI bleeding [5]. These nonspecific symptoms might delay diagnosis and treatment, causing the disease to progress and further metastasize before it is identified, leading to poorer outcomes. One study found 81% of their patients had other metastases at the time of diagnosis [4]. Furthermore, metastasis to the stomach can occur up to 10 years after the initial cancer diagnosis, distracting from timely diagnosis. Taal *et al.* also highlighted the poor prognosis associated with this rare presentation, with most patients receiving only palliative care and a 2-year survival of 23% [5]. In most reported cases, patients with gastric metastasis from breast cancer had a limited survival time following diagnosis, and the role of surgery was minimal, largely restricted to palliative bypass procedures or stenting in cases of obstruction [6–13].

Surgical intervention is rarely pursued due to the advanced nature of the disease at the time of diagnosis. In most cases, the median survival time was 10 months despite systemic therapies [5]. Standard treatment options for metastatic breast cancer include endocrine therapy, chemotherapy, and targeted therapies, such as HER2 inhibitors or CDK4/6 inhibitors [5, 9, 14–16]. In patients with extensive metastatic disease, the focus often shifts to symptom management and palliative care, especially in cases where the disease progresses after first-line therapies [5, 17].

In the case presented, the decision to proceed with a total gastrectomy in the setting of perforated metastatic gastric cancer was challenging. Without the definitive gastric resection, the patient would most certainly succumb to her sepsis from the ongoing gastric leak and peritonitis. To our knowledge, this is the first reported case where a patient with gastric metastasis from ILC survived following R1 resection in the setting of an acute perforation.

While this case highlights the potential for surgical intervention in select cases, it remains an exception rather than the rule. The general prognosis for gastric metastasis from breast cancer remains poor, and palliative surgical intervention should only be considered after considering patient’s wishes and multidisciplinary team discussion. A high-risk palliative resection was performed for our patient because she was an otherwise fit and well farmer who potentially could have second-line chemo/immunotherapy for her metastatic ILC.

In addition, this case opens the door to further discussions on treatment strategies for patients with metastatic breast cancer. There may be potential for future research into second- or third-line therapies, particularly for those who experience progression after initial treatments. As new therapies continue to evolve, including novel targeted therapies, surgery can be used to improve survival outcomes even in aggressive presentations like this one.

In conclusion, we demonstrated that emergency R1 gastrectomy for gastric perforation from metastatic ILC gastric cancer can be considered in selected cases after multidisciplinary discussion. This case adds to the limited body of literature and suggests that palliative surgical management can be lifesaving in acute complicated presentations in this subgroup of patients.

## References

[1] Australian Institute of Health and Welfare. Australia’s health 2024: in brief. Canberra: AIHW; 2024. p. 19. (Cat. no. AUS249). Available from https://www.aihw.gov.au/reports/australias-health/australias-health-2024-in-brief/summary

[2] Limaiem F, Khan M, Lotfollahzadeh S. Lobular Breast Carcinoma. [Updated 2023 Jun 3]. In: StatPearls [Internet]. Treasure Island (FL): StatPearls Publishing; 2024. Available from: https://www.ncbi.nlm.nih.gov/books/NBK554578/. 32119465

[3] Namikawa T, Munekage E, Ogawa M, et al. Clinical presentation and treatment of gastric metastasis from other malignancies of solid organs. Biomedical Reports 2017;7:159–62. 10.3892/br.2017.943 28804629 PMC5526074

[4] D’Angelo F, Rampini A, Cardella S, et al. Breast cancer metastasis to the stomach. Journal of Cancer Metastasis and Treatment 2019;5:5.

[5] Taal BG, Peterse H, Boot H. Clinical presentation, endoscopic features, and treatment of gastric metastases from breast carcinoma. Cancer. 2000;89:2214–21. 10.1002/1097-0142(20001201)89:11<2214::AID-CNCR9>3.0.CO;2-D 11147591

[6] Pectasides D, Psyrri A, Pliarchopoulou K, et al. Gastric metastases originating from breast cancer: report of 8 cases and review of the literature. Anticancer Res 2009;29:4759–63. 20032432

[7] Ayantunde AA, Agrawal A, Parsons SL, et al. Esophagogastric cancers secondary to a breast primary tumor do not require resection. World J Surg 2007;31:1597–601. 10.1007/s00268-007-9099-y 17578645

[8] Villa Guzmán JC, Espinosa J, Cervera R, et al. Gastric and colon metastasis from breast cancer: case report, review of the literature, and possible underlying mechanisms. Breast Cancer: Target and Therapy 2017;9:1–7. 10.2147/BCTT.S79506 PMC5207330 28096693

[9] Critchley AC, Harvey J, Carr M, et al. Synchronous gastric and colonic metastases of invasive lobular breast carcinoma: case report and review of the literature. Ann R Coll Surg Engl 2011;93:e49–50. 10.1308/147870811X582800 21943448 PMC5827216

[10] Weigt J, Malfertheiner P. Metastatic disease in the stomach. Gastrointest Tumors 2015;2:61–4. 10.1159/000431304 26674003 PMC4668793

[11] Namikawa T, Hanazaki K. Clinicopathological features and treatment outcomes of metastatic tumors in the stomach. Surg Today 2014;44:1392–9. 10.1007/s00595-013-0671-9 23896636

[12] Jones GE, Strauss DC, Forshaw MJ, et al. Breast cancer metastasis to the stomach may mimic primary gastric cancer: report of two cases and review of literature. World J Surg Oncol 2007;5:75. 10.1186/1477-7819-5-75 17620117 PMC1937002

[13] Aurello P, D'Angelo F, Cosenza G, et al. Gastric metastasis 14 years after mastectomy for breast lobular carcinoma: case report and literature review. Am Surg 2006;72:456–60. 10.1177/000313480607200518 16719204

[14] Reiman T, Butts CA. Upper gastrointestinal bleeding as a metastatic manifestation of breast cancer: a case report and review of the literature. Can J Gastroenterol 2001;15:67–71. 10.1155/2001/898434 11248910

[15] Karamlou TB, Vetto JT, Corless C, et al. Metastatic breast cancer manifested as refractory anemia and gastric polyps. South Med J 2002;95:922–5 2-s2.0-0036340636. 10.1097/00007611-200295080-00027 12190233

[16] Gerova VA, Tankova LT, Mihova AA, et al. Gastrointestinal metastases from breast cancer: report of two cases. Hepato-Gastroenterology. 2012;59:178–81. 10.5754/hge10681 22251535

[17] Taal BG, den Hartog Jager FCA, Steinmetz R, et al. The spectrum of gastrointestinal metastases of breast carcinoma: I. Stomach Gastrointestinal Endoscopy 1992;38:130–5. 10.1016/S0016-5107(92)70377-0 1568608

